# Incidence and risk factors for postsurgical gastroparesis syndrome after laparoscopic and open radical gastrectomy

**DOI:** 10.1186/1477-7819-11-144

**Published:** 2013-06-19

**Authors:** Hongbo Meng, Donglei Zhou, Xun Jiang, Weixing Ding, Liesheng Lu

**Affiliations:** 1 Department of General Surgery, Tenth People’s Hospital of Tongji University, Shanghai 200072, China

**Keywords:** Laparoscope, Radical gastrectomy, Gastroparesis, Psychological factors

## Abstract

**Background:**

The aim of this study was to investigate the differences and influencing factors for postsurgical gastroparesis syndrome incidence after laparoscopic and open radical gastrectomy.

**Methods:**

Clinical data were collected for 563 patients who underwent open radical gastrectomy for gastric cancer and 72 cases receiving laparoscopic radical gastrectomy. We retrospectively analyzed the incidence of postsurgical gastroparesis syndrome, clinical features, course of disease, and risk factors of these two groups.

**Results:**

There was no statistical difference for the incident rate of postsurgical gastroparesis syndrome between laparoscopic and open radical gastrectomy (6.9% vs. 3.7%, *P* > 0.05). Preoperative outflow tract obstruction and Billroth II anastomosis were the two risk factors for postsurgical gastroparesis syndrome in the open radical gastrectomy group and the laparoscopic surgery for gastric cancer group. The same results were obtained from logistic regression statistical analysis. Age greater than 70 years was also one of the risk factors for postsurgical gastroparesis syndrome in the open radical gastrectomy group (*P* < 0.05).

**Conclusions:**

Laparoscopic radical gastrectomy for gastric cancer does not increase the incident rate of postsurgical gastroparesis syndrome.

## Background

Gastroparesis is a chronic heterogeneous disorder of gastric motility. Gastroparesis is defined as delayed gastric emptying of a solid meal in the absence of mechanical obstruction [1]. Characteristic symptoms of gastroparesis ranged from mild to severe include nausea, vomiting, epigastric pain, early satiety, fullness, anorexia, and/or weight loss [2]. Delayed gastric emptying is considered to contribute to functional dyspepsia and gastroesophageal reflux disease [3], and severely damages the ability of patients to manage nutrition, health, and social interactions. Some studies suggest there are many etiologies for gastroparesis. The underlying cause cannot be identified in 36 to 49% of patients [4], which is called idiopathic gastroparesis.

At present, increasing interest has led to dramatic increases in the characterization and diagnosis of gastroparesis; however, the disease remains under-recognized [5]. When gastroparesis is appropriately diagnosed, medical therapy can relieve some of its symptoms [6]. The commonly used medicines are prokinetic agents, such as metoclopramide, erythromycin, domperidone, and cisapride. A large number of patients find it difficult to tolerate long-term treatment due to the side effects of these drugs, including the drug-refractory, large economical burden and great psychic pain for patients. Because the efficacy of medicine in the therapy of gastroparesis is certainly limited, gastroparesis also brings continuing huge challenges for physicians.

Open and laparoscopic radical gastrectomy are the routine treatments for gastric cancer. The common feature of the two surgeries is detrimental to normal anatomical physiology function, which can lead to different degrees of disorders of the gastrointestinal motility, especially gastric emptying dysfunction. Some patients cannot return to normal, which can result in prolonged postoperative recovery time and hospital stays, and increasing psychological burden. The aim of this study was to retrospectively analyze the incidence of postsurgical gastroparesis syndrome among gastric carcinoma patients receiving open radical gastrectomy from January 2004 to January 2010 and receiving laparoscopic radical gastrectomy from April 2009 to December 2010 and to explore their clinical features, course of disease and risk factors.

## Methods

This retrospective study was approved by the ethics committee of Tenth People’s Hospital of Tongji University and written informed consent was obtained from all patients receiving treatment. From January 2004 to January 2010, 563 patients underwent open radical gastrectomy for gastric cancer (standard D2 lymphadenectomy). From April 2009 to December 2010, 72 patients received laparoscopic radical gastrectomy for gastric cancer (standard D2 lymphadenectomy). All cases were diagnosed according to the following criteria: one or multiple tests suggesting there was no mechanical obstruction in the gastric outflow tract; the drainage volume from the stomach was > 800 ml/day and lasted for more than 10 days; there was no obvious water–electrolyte imbalance; gastroparesis was not induced by conditions such as diabetes, hypothyroidism and connective tissue disease; and no drugs affecting contraction of smooth muscle were used.

All cases were examined by upper gastroenterography with 30% meglumine diatrizoate and a gastroscope during the diagnosis and treatment process. Suspected gastroparesis patients without upper gastroenterography and gastroscope examination were excluded. The fasting plasma albumin and blood glucose were detected before operation. All gastroscope examinations showed that there were no peristaltic waves or few peristaltic waves, or chronic inflammation or mild ulcer of anastomosis, and no mechanical obstruction in gastroenteric anastomosis passed by endoscopy. Upper gastroenterography with oral meglumine diatrizoate observing a dynamic change of contrast agent in the stomach showed that there were weak peristaltic waves or no peristaltic waves; obviously, residual contrast agent in the stomach indicated no mechanical obstruction in gastroenteric anastomosis and delayed gastric emptying.

Combining with the literature [7,8], we compared the open operation group with the laparoscopic surgery group in terms of age, gender, surgical procedures, obstruction of the outflow track, and albumin level before operation. All statistical analyses were performed with SPSS 17.0 software (SPSS Inc., Chicago, IL, USA). The chi-square test was used to compare the incident rate for gastroparesis for the two groups. The logistic regression method was employed to analyze the risk factors for the incident rate of gastroparesis postoperatively.

## Results

Twenty-one patients (12 males, nine females; average age 69.8 years) developed gastroparesis in the open operation group with an incident rate of 3.7% (21/563). Five cases had discomfort epigastric satiety and recurrent vomiting from a liquid to semifluid diet 6 to 7 days postoperatively. Physical examination revealed capotement. The drainage volume in the other 16 patients gradually increased and gastrointestinal decompression continued until the recovery of gastric motility. Five patients (four males, one female; average age 71.3 years) developed gastroparesis in the laparoscopic surgery group with an incident rate of 6.9% (5/72). One patient had discomfort epigastric satiety and recurrent vomiting from a liquid to semifluid diet 7 days postoperatively. Other patients showed that the drainage volume gradually increased during indwelling gastric tube use postoperatively and gastrointestinal decompression was maintained until the recovery of gastric motility. There was no statistical difference for gastroparesis incidence between the two groups (6.9% vs. 3.7%, *P* = 0.197).

We conducted a correlation analysis between the gastroparesis incidence and different risk factors such as age, gender, hypoalbuminemia before operation, outflow track obstruction, and the method of gastroenteric anastomosis. The statistical analysis in the open operation group showed that the incident rate of gastroparesis at > 70 years old was obviously higher than that at < 70 years old. The occurrence of gastroparesis in Billroth II operation was markedly higher than that in Billroth I operation. Gastroparesis incidence in patients with outflow track obstruction before surgery was significantly higher than that in those patients without outflow track obstruction. Gastroparesis occurrence was not associated with gender and hypoalbuminemia before the surgery (see Table 1). The statistical results of the laparoscopic operation group suggested that Billroth II anastomosis and outflow track obstruction before the surgery were the risk factors for gastroparesis (see Table 2). Therefore, the gastroparesis incidence after radical subtotal gastrectomy was significantly related to the outflow track obstruction before surgery and the method of reconstruction of the gastrointestinal tract.

**Table 1:** Correlation between the gastroparesis incidence and different factors after open radical gastrectomy for gastric cancer

**Group**	**Age (>70/<70 years)**	**Gender (male/female)**	**Surgery type (Billroth I/Billroth II)**	**Outflow track obstruction (yes/no)**	**Hypoalbuminemia (yes/no)**
Gastroparesis group	16/5	12/9	7/14	13/8	7/14
Nongastroparesis group	299/253	301/251	306/246	211/341	187/365
χ^2^ value	3.964	0.056	3.987	4.794	1.231
*P* value	0.046	0.813	0.045	0.029	0.759

**Table 2:** Correlation between the gastroparesis incidence and different factors after laparoscopic radical gastrectomy for gastric cancer

**Group**	**Age (>70/<70 years)**	**Gender (male/female)**	**Surgery type (Billroth I/Billroth II)**	**Outflow track obstruction (yes/no)**	**Hypoalbuminemia (yes/no)**
Gastroparesis group	4/1	4/1	0/5	4/1	1/4
Nongastroparesis group	41/26	39/28	43/24	20/47	23/44
*P* value	0.644	0.642	0.008	0.039	0.659

*P* value calculated by Fisher’s exact probability method (two-tail).

Further, nonconditional and multifactorial logistic regression analysis was conducted with gastroparesis incidence as the dependent variable and other statistics as independent variables. The results also showed that outflow track obstruction before surgery and Billroth II anastomosis were the two risk factors. Laparoscopic surgery did not increase the risk of gastroparesis incidence (see Table 3).

**Table 3:** Results of multifactorial nonconditional logistic regression analysis

**Relevant factor**	**Odds ratio**	**Chi-square**	***P *value**
Outflow track obstruction before surgery	3.179	8.967	0.018
Billroth II anastomosis	4.278	7.657	0.008

All gastroparesis patients were provided with continuing gastrointestinal decompression, fluid infusion, and nutritional support to maintain water–electrolyte balance. The patients with hypoalbuminemia were supported with human albumin. Metoclopramide 20 mg/day intravenously, domperidone 10 mg three times daily by stomach tube injection, and erythromycin 250 mg twice daily by stomach tube injection became routine treatments. Some patients were given acupuncture, physiotherapy and other therapies at the same time Twenty-one gastroparesis patients in the open operation group and five cases in the laparoscopic surgery group recovered gastric motility and were successfully discharged after the conservative treatment. Among these patients, six cases recovered gastric motility within 2 weeks, 10 cases within 3 weeks, eight cases within 4 weeks, and two cases within > 4 weeks. There were no patients who underwent reoperation The recommendations for psychotherapy were as follows: we consulted the psychologist in our hospital, worked out courses of psychological counseling and educated patients with the help of nurses and family.

## Discussion

Gastroparesis is a disorder of the gastrointestinal tract with variable manifestations. Gastric scintigraphy has been widely used for the diagnosis of gastroparesis. However, symptom severity and clinical presentation do not relate to the degree of delay in gastric emptying [9,10]. Unlike gastroesophageal reflux disease, where erosive esophagitis, esophageal peptic stricture and Barrett’s esophagus are specific identifiers for patients with severe disease, the clinical features that indicate the severity of gastroparesis are poorly defined [11]. Symptoms of upper abdominal pain, nausea, and vomiting are subjective, and objective markers for complications of severe gastroparesis are lacking [11]. Gastroparesis may stem from neuromuscular dysfunction. Diabetic gastroparesis is ranked by more than two-thirds of respondents as the main cause of gastroparesis. The prevalence for the causes of gastroparesis is unclear because of limited epidemiologic data. Clinical presentation of gastroparesis is very varied. The results of one population study from Olmstead County, Minnesota show that nausea and vomiting are common (74% and 53%, respectively) compared with abdominal pain (45%) [4].

Recently, with the prevalent and improved technique of laparoscopy, radical operation for carcinoma of the stomach under laparoscopy (standard D2 lymphadenectomy) with a series of advantages such as a small wound and early recovery is more popular. However, at present there are few studies about gastroparesis after laparoscopic radical gastrectomy for gastric cancer. This study analyzed retrospectively the incidence and influencing factors for postsurgical gastroparesis syndrome after laparoscopic and open radical gastrectomy. Gastroparesis is one complication in abdominal surgeries and its incidence has a gradually increasing trend. The main clinical manifestation of gastroparesis is nonmechanical obstruction of the gastric outflow track. The mechanism of gastroparesis so far still remains unclear. Gastroparesis may associate with damage of a gastric electrical pacemaker, gastrointestinal dysfunction, and vagus resection after subtotal gastrectomy [12] Multiple factors may be attributable to gastroparesis incidence, and the interaction or interference among varying factors may make false sense. The results of this study showed that there was no statistical difference for gastroparesis incidence between open and laparoscopic surgery (*P* > 0.05). In terms of absolute incidence, however, gastroparesis occurrence from open was lower than that from laparoscopic surgery (3.7% vs. 6.9% ), which may be associated with the small sample size in the laparoscopic surgery group. The results from statistical analysis in the open operation group and the laparoscopic surgery group, respectively, suggested that preoperative outflow tract obstruction and Billroth II anastomosis were the two risk factors for postsurgical gastroparesis syndrome (*P* < 0.05). In the open operation group, age was also a risk factor for gastroparesis occurrence, while there was no statistical difference for age in the laparoscopic surgery group. The small number of patients in the laparoscopic surgery group may be a critical factor contributing to this difference. Increasing the sample size of the laparoscopic surgery group is therefore needed for further study.

The main difference between laparoscopic and open radical operation for carcinoma of the stomach is the operation time except for the operative incision and surgical trauma In this study, there is no statistical analysis for operation time between the two groups; however, the operation time for radical correction under the laparoscope for gastric cancer is longer than that from open radical operation. The time of every operation was over 240 minutes in the five gastroparesis patients of the laparoscopic group, among which the longest time was 320 minutes. Among these five patients there are three patients with previous surgeries, which can cause serious intraperitoneal adhesion and prolong the operation time. In addition, lymphatic cleaning, the stomach being pulled excessively during the operation, and too much ligation and damage of gastric vessels can cause injuries of the gastric smooth muscle, gastric nerve plexuses and retroperitoneal nerve plexuses, leading to delayed gastric emptying [13] Old age is also a factor for increased operation difficulty, owing to increased tissue brittleness, easy bleeding, body functions inferior to young patients and accompanying cardiopulmonary disease. The patients with basic disease, a serious condition and previous other abdominal surgeries may therefore have a longer operation time, and we should carefully assess these patients preoperatively, control the basic disease and improve the condition of the whole body. A newly established surgical team, unfamiliar surgical assistants and deficient cooperation also became factors for the prolonged operation time in five gastroparesis patients of the laparoscopic group. The improvement of the professionalism and technical proficiency and level of the surgeon, especially the assistant, and the sensible division of surgeons participating in the operation may be beneficial for gastroparesis prevention, and we should work closely with the operator to make operations orderly and shorten the operation time.

The psychology factor may play an important role in the incidence and treatment of gastroparesis. Stress reaction of the organism caused by anxiety, nervousness, fear, insomnia, and so on, in the perioperative period makes the vegetative nervous system function disordered, excites the sympathetic nerve, and inhibits the activated neurons of gastrointestinal nerves plexus and catecholamine released by the sympathetic nerve ending to directly combine the α-receptor and β-receptor in the cytomembrane of gastrointestinal smooth muscle cells, and the contraction of gastrointestinal smooth muscle cells and gastric emptying [14]. This study showed that there were serious psychological anxieties existing in five gastroparesis patients from the laparoscopic surgery group, who paid great attention to the disease before and after operation, focused on even the smallest details, imagined the seriousness of their own disease, and always considered themselves not well when diagnosing gastroparesis. In those patients, gastric juice could not reduce and there were certain degrees of psychological hints. The treatment of the gastroparesis patient with most serious psychological anxieties lasted 6 months. The patient was forcibly removed from the gastric tube under the condition of gastric juice 1,000 ml/day and then provided with psychic solace, and after 3 days successfully took food without feeling unwell. These findings indicate that the psychological factor is an important factor for incidence of gastroparesis.

Basic existing diseases are a risk factor for delayed gastric emptying. Hyperglycemia can inhibit the secretion and release of motilin. Blood sugar > 10 mmol/l can induce electrogastric dysrhythmias and reduce intragastric pressure, and finally delay gastric emptying. Hyperglycemia can also inhibit the promoting action of erythrocin for gastric emptying. Hypoproteinemia after the operation can easily induce edema of gastrointestinal walls and anastomosis, resulting in local dyskinesia and prolonging the recovery of gastrointestinal function. Although this study eliminates the patients with a clear diagnosis of diabetes, some patients included still have temporal hyperglycemia after the surgery. Moreover, patients are in the state of bearing cancer, and some patients still have pyloric obstruction with undernutrition and hypoproteinemia or anemia. Those factors increase the incidence of gastroparesis. However, the results of the study suggest that hypoproteinemia is not a risk factor for gastroparesis incidence.

We believe the following measures play certain roles in preventing gastroparesis incidence. Blood sugar should be actively controlled before surgery. Hypoproteinemia and anemia should be corrected. Enough calories should be supported after the operation. An appropriate amount of blood, plasma, and albumin can be transfused. Glucose transfusion should be controlled during the fluid infusion. Enteral nutrition is provided to the greatest extent. Chyme normally passes the stimulation at the initial part of the duodenum from the stomach after Billroth I anastomosis, which accords with normal anatomy and physiology and coordinates the gastrointestinal motor. As a result, in patients with Billroth I anastomosis the recovery of gastrointestinal function after operation is rapid and the gastrointestinal motor coordinates normally, while the patients are prone to gastroparesis incidence, owing to an obvious change of gastric conditions, and spasm and poor coordination of peristole [14]. The results of this study accord with that of previous research. The reconstruction process of the gastrointestinal track conforms to the state of anatomy and physiology to the greatest extent, which leads to gastrointestinal motor coordination. Billroth I anastomosis should be attempted if conditions permit in patients with benign disease.

## Conclusion

In brief, gastroparesis after radical operation for gastric cancer is not common; however, it is still worthy of concern. This study suggests there is no statistical difference for the incident rate of postsurgical gastroparesis syndrome between laparoscopic and open radical gastrectomy, and psychological therapy may play an important role in the treatment of gastroparesis, which provides new ideas for gastroparesis research.

## Competing interests

The authors declare that they have no competing interests.

## Authors’ contributions

HM and DZ participated in the design, analyses and data interpretation and drafted the manuscript. XJ, WD and LL helped to retrieve pathologic and clinical information and provide valuable insight during manuscript preparation. All authors reviewed and approved the final manuscript.

## Authors’ information

Hongbo Meng and Donglei Zhou should be regarded as co-first authors.
